# Incidence, risk factors and outcomes of cataract surgery after plaque brachytherapy for posterior uveal melanoma

**DOI:** 10.1016/j.heliyon.2023.e23447

**Published:** 2023-12-09

**Authors:** Viktor T. Gill, Gustav Stålhammar

**Affiliations:** a Department of Pathology, Västmanland Hospital Västerås, Västerås, Sweden; b Department of Clinical Neuroscience, Division of Eye and Vision, Karolinska Institutet, Stockholm, Sweden; c Ocular Oncology Service and St. Erik Ophthalmic Pathology Laboratory, St. Erik Eye Hospital, Stockholm, Sweden

## Abstract

**Purpose:**

To examine incidence, risk factors, and outcomes of cataract surgery after plaque brachytherapy for posterior uveal melanoma.

**Design:**

Retrospective interventional cohort study contrasted with general population data.

**Methods:**

All patients treated with plaque brachytherapy for a posterior uveal melanoma at Sweden's national referral center between 2010 and 2022 were included (*n* = 933). These patients were cross-referenced with data from the Swedish National Cataract Register. Competing risk incidences and outcomes of cataract surgery were compared with a random sample of 1000 individuals from the general population.

**Results:**

The 12-year incidence of cataract surgery after plaque brachytherapy was 27 % (95 % CI 23–31 %), which markedly exceeded the incidence of 16 % in the general population (95 % CI 13–18 %, Gray's *P* < 0.001). Patients treated with Iodine-125 had significantly higher incidence than patients treated with Ruthenium-106, and the latter had greater incidence than the general population (*P* < 0.001). In univariate competing risk regressions, older patients, female sex, thick tumors, and Iodine-125 were associated with cataract surgery. In multivariate analysis, older patients and Iodine-125 retained their significance. Outcomes of cataract surgery were overall similar in the plaque brachytherapy and general population, but the general population more often received post op. topical NSAID.

**Conclusions:**

In this study, plaque brachytherapy for posterior uveal melanoma was associated with a significantly increased incidence of cataract surgery. Treatment with the gamma emitting isotope iodine-125 and older patient age at the time of brachytherapy emerged as the major risk factors. Outcomes of cataract surgery were comparable to the general population.

## HIGHLIGHTS

• A large retrospective long-term follow-up cohort of uveal melanoma patients.
• 27 % incidence: Cataract surgery post-brachytherapy vs. 16 % in general population.
• Iodine-125 risk: 2.5× cataract surgery incidence compared to general population.
• Ruthenium-106 risk: 1.3× incidence.
• Aging factor: Older age at brachytherapy ups cataract surgery risk.

## 1 Introduction

Radiation therapy, commonly used to treat ocular tumors, frequently results in post-treatment cataracts. Among the numerous available treatment modalities, including particle irradiation proton therapy, and external beam techniques like stereotactic radiotherapy and robotic radiosurgery, ophthalmic plaque brachytherapy stands out as the most common eye-preserving approach for treating posterior uveal melanoma (UM) [[1], [2], [3], [4]]. While various radioisotopes are in use, iodine-125 and ruthenium-106 plaques are adapted worldwide [[5], [6], [7], [8], [9], [10], [11], [12]]. The latter, owing to its steep β radiation dose fall-off curve, is preferred for tumors less than 6 mm in apical thickness [13,14]. Despite these techniques typically ensuring acceptable local treatment failure rates and comparable patient survival rates to enucleation, the side effects of radiation, such as retinopathy, neuropathy, maculopathy, and cataract, remain significant considerations due to their potential impact on patients’ vision-related quality of life [15,16].

While modern cataract surgery is associated with minimal risks, it remains an invasive procedure. Patients may experience severe anxiety if they don't understand the reason for a declining visual acuity. Depending on how they have been informed, they might attribute it to radiation damage to the retina or fear a tumor recurrence. Some patients are hesitant about facing another surgery following brachytherapy, or about the vision-restoring potential in an eye damaged by tumor growth and therapy. For these reasons, and the great impact cataract can have on daily activities like driving, it is paramount for clinicians to understand the incidence, risks, and outcomes post-brachytherapy. While radiation dose has been scrutinized as a cataractogenesis biomarker, factors such as patient age, systemic health conditions, and tumor attributes are equally pivotal in understanding post-radiation cataract onset [17,18].

This study delves deeper into these aspects, utilizing a larger cohort with longer follow-up. We meticulously analyzed 933 UM patients treated with plaque brachytherapy, comparing them to a 1000-person sample from the general population. Our data, sourced from the nation's sole ocular oncology center and cross-referenced with the national cataract register, revealed that patients treated with iodine-125 had a 2.5-fold incidence of cataract surgery to that of the general population. Patients treated with ruthenium-106 had a 1.3-fold incidence. Patient age emerged as the second key risk determinant, whereas tumor thickness and tumor location were not independent predictors in multivariate competing risk regressions. Intriguingly, cataract surgery outcomes remained consistent between the study cohort and the broader population.

## 2 Methods

### 2.1 Patients and samples

All 933 patients treated for posterior UM (involving the choroid and/or ciliary body) with ruthenium-106 or Iodine-125 plaque brachytherapy at St. Erik Eye Hospital, Stockholm, Sweden between January 1st, 2010, and December 31st, 2022, were included in the study. Patients with iris melanoma were excluded.

Throughout the period, St. Erik Eye Hospital was the only institution in Sweden to treat UM with plaque brachytherapy, ensuring complete coverage of all patients. Ruthenium plaques and Iodine seeds were delivered by Eckert & Ziegler BEBIG, Berlin, Germany, and source specification data from the manufacturer were independently verified by medical physicists at the Karolinska University Hospital, Stockholm. All primary tumors were diagnosed by ocular oncologists by slit-lamp biomicroscopy, indirect ophthalmoscopy, A- and B-scan ultrasonography, fundus imaging, and optical coherence tomography (OCT), as needed. Transvitreal biopsies were performed if a diagnosis could not be established from the clinical examination.

Plaque brachytherapy with ruthenium-106 plaques was typically reserved for tumors with an apical thickness of <6 mm, with thicker tumors being treated with iodine-125. Tumors with an apical thickness of >10 mm were treated with enucleation. Extrascleral extension was also an indication for enucleation, unless minimal. Tumor proximity to the optic disc (e.g., <2 mm or similar) was not an absolute contraindication for brachytherapy, as reported previously [19]. The brachytherapy procedure was performed under general anesthesia.

Clinicopathological and follow-up data including secondary enucleation and mortality was collected from our treatment register. The study adhered to the tenets of the Declaration of Helsinki, and approval was obtained from the Swedish Ethical Review Authority (reference 2022-00930-02). Informed consent was waived because this was a retrospective chart review that did not influence patient management (i.e., treatment, testing, follow-up, or information to patients), did not include any analyses of biological tissues, and was based on already collected data.

### 2.2 Power calculation

A post-hoc power calculation was conducted. For the 933 UM patients, we estimated a 12-year competing risk incidence of cataract surgery of 40 %. This estimate is informed by a prior study's 8-year figures, with a cumulative incidence of just over 50 % after plaque brachytherapy of large tumors (which should lead to a higher incidence than treatment of smaller tumors) [20]. While definitive 10 or 12-year incidence data for age-matched individuals in the general population is unavailable, we extrapolated a rough approximate rate of 30 % from a Swedish study that reported a 1-year cataract surgery rate of 2–6 per 100 for those aged 70 and above [21]. Based on an assumed 10 % incidence difference (40 % vs. 30 %), our study achieves a power of >99.5 % at an α of 0.05, provided that the comparison group was *n* ≥ 950.

### 2.3 General population sample

We collected a comparison sample of 1000 individuals from the general population alive as of January 1st, 2010. The population register, integrated into our digitalized medical record system, was the source for this sampling. For a balanced comparison, the general population sample was matched to the UM cohort based on sex and birth year. Specifically:

● *Sex*: The general population sample mirrored the sex distribution of the UM patients within a margin of ±1% point.
● *Birth Year*: For each birth year, the number of individuals selected from the general population was set to mirror the melanoma cohort, allowing for a variance of up to 5 individuals. To illustrate, if there were 20 patients from the melanoma group born in 1955, we would select anywhere from 15 to 25 individuals born in 1955 from the general population who were still alive in 2010.

By cross-checking with the National Cataract Register, we could identify which of these 1000 individuals had undergone cataract surgery, but no medical records or personal identifiers were collected, including personal identity numbers, names, addresses, contact information, or photographs.

### 2.4 Swedish National Cataract Register

The UM patients as well as the individuals in the general population sample were crosschecked with data from the Swedish National Cataract Register (NCR) for the years 2010 through 2022. NCR has been estimated to capture 93 % of all cataract surgeries in the country. The variables in the core register for all cataract procedures have been published, and their changes over the years since the foundation in 1992 are reported in the Swedish meta-database Register Utilization Tool through the Swedish Research Council [22]. For all matches in the NCR, we collected data on the date of cataract surgery, best corrected visual acuity (BCVA) pre- and post cataract surgery (of the tumor eye for UM patients, of the first eye undergoing cataract surgery for individuals from the general population), refractive surprise, details on the surgery including lens material and color, co-existing conditions at the time of cataract surgery such as glaucoma and pseudo exfoliations, and on the use of topical medications.

### 2.5 Visual acuity and follow-up

After plaque brachytherapy, patients underwent routine ophthalmological assessment at 1, 3, 6, and 12 months. If local control had been achieved, the patient was then seen semiannually or annually until death. At each visit, patients were examined with slit-lamp biomicroscopy and A- and B-scan ultrasonography. Fundus photos, OCT, and other imaging studies were added, as needed. Intraocular pressures (IOP) and BCVA were measured before plaque brachytherapy and then at each follow-up visit, as described previously [23]. The BCVA recorded was the smallest line at which five of five or six of seven letters were correctly identified after subjective refraction and corrected in a trial frame. All analyses related to VA were performed using the LogMAR scale, converted to Snellen equivalents and decimal scale for display only. Poor BCVA was defined as LogMAR ≥1.00, following a previously used classification [23,24].

### 2.6 Statistical methods

Differences with a *P* < 0.05 were considered significant, and all *P* values were two-sided. The Shapiro‒Wilk test was used to evaluate the deviation of continuous variables from a normal distribution. If the test was significant (*P* < 0.05), the Mann‒Whitney *U* test was used to compare groups; otherwise, Student's *t*-test was used. Pearson's chi-square (χ^2^) test (if all fields had a sample of ≥5) or Fisher's exact test (F, if any field had a sample of <5) was used in contingency tables. For comparisons of BCVA before and after cataract surgery, the Wilcoxon matched-pairs signed rank test was used. Competing risk regressions with 95 % confidence interval (CIs) were used to analyze associations with cataract surgery [25]. All significant predictors from univariate competing risk regressions were included in multivariate analyses. Additionally, any variables that differed with a *P* < 0.10 in descriptive statistics of UM patients vs. the general population sample were included as covariates in multivariate regressions. In comparisons of thin versus thick tumors, we used the median value for tumor apical thickness, rounded to the nearest integer as cutoff (i.e., 5.0 mm). To determine whether our follow-up data met the proportional hazard assumption, we used a graphical approach and assessed log-minus-log-transformed survival curves for time to cataract surgery. If survival curves were parallel without any crossing or divergence, the proportional hazard assumption was considered fulfilled. Cumulative incidence function estimates from competing risk data were plotted with the cmprsk package for R, and the equality of survival distributions was tested with Gray's test for equality [26]. All statistical analyses except competing risk survival analyses were performed using IBM SPSS statistics version 27 (Armonk, NY) and GraphPad Prism version 9.3.0 (San Diego, CA, USA).

## 3 Results

### 3.1 Descriptive statistics

Among the 933 UM patients, 446 (48 %) were female. The average age at the time of UM diagnosis was 65 years, with a standard deviation (SD) of 13 years. The BCVA in the affected eye was LogMAR 0.68 (SD 0.59). The average largest basal tumor diameter (LBD) was 10.6 mm (SD 3.5 mm) while the mean tumor thickness was 5.1 mm (SD 2.6 mm).

**Fig. 1:**
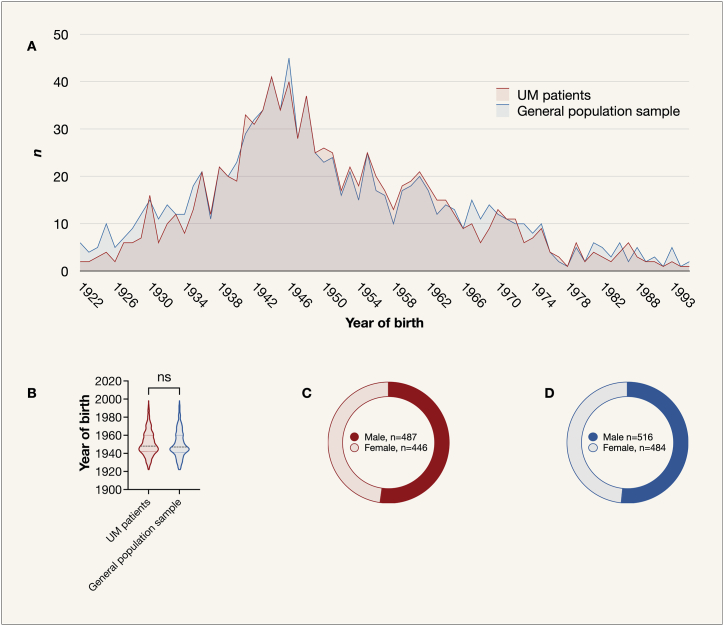
Distribution of birth year and sex in the UM cohort and general population sample. The general population sample was matched to the UM cohort based on sex and birth year, and the year of birth was similarly distributed between the two groups when tested as A) a categorical variable (χ^2^*P* > 0.99), and B) as a continuous variable (Mann-Whitney *U P* = 0.13). Females represented 48 % of both C) UM patients, and D) the general populations sample (χ^2^*P* = 0.79). χ^2^, Chi-Square test. UM, uveal melanoma.

Birth years deviated from a normal distribution for both UM patients (Shapiro-Wilk test *P* < 0.001) and the general population sample (*P* < 0.001). The distribution of patient sex was similar between UM patients and the general population (χ^2^ *P* = 0.79). The year of birth was also similarly distributed between the two groups (χ^2^ *P* > 0.99; Mann-Whitney *U P* = 0.13, Fig. 1).

A cross-reference was made between the 933 UM patients and 1000 individuals from the general population sample with data on 1,515,983 cataract surgeries conducted from 2010 to 2022 (see Supplementary Table 1). By the time of data collection, 258 UM patients and 187 from the general sample had undergone cataract surgery. Eighty-six of the 258 UM patients (33 %) had undergone their cataract surgery before plaque brachytherapy (mean −3.1 years, SD 2.8), and these were excluded from analyses of the cumulative incidence and outcomes of cataract surgery after plaque brachytherapy. Mortality figures (from any cause) were 193 for the UM patients and 142 for the general population. Additionally, 27 UM patients underwent secondary enucleation (after primary plaque brachytherapy). Our data for time to cataract surgery met the proportional hazards assumption, as indicated by non-crossing or divergent log-minus-log-transformed survival curves (Supplementary Fig. 1). Additional details on clinicopathological characteristics, treatment modalities, and follow-up parameters can be found in Table 1.

**Table 1:** Characteristics of included patients, tumors, and treatment variables.

	UM brachytherapy patients, *n* = 933	General population sample, *n* = 1000	*P*
**Sex, *n* (%)**			0.79*
Female	446 (48)	484 (48)	
Male	487 (52)	516 (52)	
**Age on January 1**st**, 2010, median years (IQR)**	62 (18)	63 (19)	0.13^†^
**Age at uveal melanoma diagnosis, mean years (SD)**	65 (13)		
**BCVA**^a^**, mean LogMAR (SD)**	0.68 (0.59)		
**Tumor LBD, mean mm (SD)**	10.6 (3.5)		
**Tumor thickness, mean mm (SD)**	5.1 (2.6)		
**Tumor distance to OD, mean mm (SD)**	4.0 (3.5)		
**AJCC T-category, *n* (%)**			
T1a	375 (40)		
T1b	29 (3)		
T1c	2 (<1)		
T2a	289 (31)		
T2b	32 (3)		
T2c	2 (<1)		
T3a	145 (16)		
T3b	43 (5)		
T3c	1 (<1)		
T4a	7 (1)		
T4b	8 (1)		
**AJCC stage, *n* (%)**			
I	375 (40)		
IIA	320 (34)		
IIB	177 (19)		
IIIA	53 (6)		
IIIB	8 (1)		
**Prescribed dose to tumor apex**^b^**, mean Gy (SD)**	94 (13)		
**Dose rate at tumor apex, mean Gy/h (SD)**	1.4 (0.7)		
**Scleral dose, mean Gy (SD)**	448 (282)		
**Adjunct TTT**^c^**, *n* (%)**	155 (17)		
**Median follow-up years (IQR)**			
To cataract surgery	1.4 (3.7)	7.8 (6.8)	
To secondary enucleation	2.1 (2.3)	–	
To death from any cause	3.7 (3.1)	7.8 (5.0)	
Alive and cataract surgery-free	5.1 (5.6)	13.0 (0.0)	

a Best corrected visual acuity of the tumor eye prior to plaque brachytherapy.

b 100 Gy for Ruthenium-106, 85 Gy for Iodine-125.

c TTT to central tumor margins (*i.e*., closest to the optic disc and fovea) at the time of primary brachytherapy. AJCC, American Joint Committee on Cancer. BCVA, best corrected visual acuity. Gy, Gray. IQR, interquartile range. LBD, largest basal tumor diameter. OD, optic disc. SD, standard deviation. TTT, transpupillary thermotherapy. UM, uveal melanoma. *Chi-square test. ^†^Mann-Whitney *U* test.

### 3.2 Incidence versus patient, tumor, and treatment characteristics

**Fig. 2:**
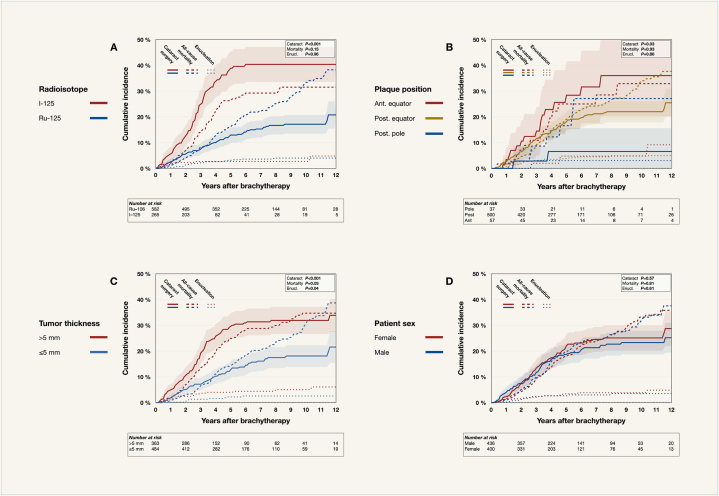
Competing risk cumulative incidence of cataract surgery, all-cause mortality, and enucleation after plaque brachytherapy for UM. Of 933 UM patients, 86 had undergone cataract surgery before plaque brachytherapy. A) By radioisotope (iodine-126 versus ruthenium-106). B) By tumor location (anterior to the equator vs. posterior to the equator vs. posterior pole. Posterior pole defined as the 6 mm diameter circular zone centered on the fovea, situated between the superior and inferior temporal arteries). C) By tumor thickness (>5 vs. ≤5 mm). D) By patient sex (female vs. male). Ant., anterior. Enucl., enucleation. I-125, iodine-125. Post., posterior. Ru-106, ruthenium-106. UM, uveal melanoma. vs., versus.

The 12-year competing risk incidence of cataract surgery for UM patients treated with Iodine-125 plaque brachytherapy was 40 % (95 % CI 34–47 %). This exceeded the 12-year incidence of 21 % for patients treated with Ruthenium-106 (95 % CI 15–26 %, Gray's *P* < 0.001). The incidence of all-cause mortality (*P* = 0.15) and secondary enucleation (*P* = 0.96) did not differ significantly between patients treated with Iodine-125 and Ruthenium-106 (Fig. 2A).

The location of the tumor in the anteroposterior dimension, and thereby the position of the plaque, was also associated with the incidence of cataract surgery. Treatment of tumors with an epicenter anterior to the eye's equator was associated with a 12-year incidence of 36 % (95 % CI 20–52 %), whereas treatment of tumors with an epicenter within the posterior pole (defined as having their epicenter within a 6 mm diameter circular zone centered on the fovea, situated between the superior and inferior temporal arteries) was associated with a 12-year incidence of only 7 % (95 % CI 0–16 %, Gray's *P* = 0.03, Fig. 2B).

Patients with thick tumors had a greater incidence of cataract surgery than those with thinner tumors: The 12-year incidence after treatment of tumors >5 mm was 34 % (95 % CI 28–40 %), and 22 % after treatment of tumors ≤5 mm (95 % CI 15–28 %, Gray's *P* < 0.001). As expected, patients with thicker tumors also had greater incidence of all-cause mortality (*P* = 0.03) and secondary enucleation (*P* = 0.04 Fig. 2C).

Patient sex was not related to the incidence of cataract surgery (Gray's *P* = 0.57), all-cause mortality (*P* = 0.81), or secondary enucleation (*P* = 0.61, Fig. 2D).

Lastly, older patient age at the time of plaque brachytherapy was associated with a greater incidence of both cataract surgery and all-cause mortality. For patients ≤30 years at the time of brachytherapy, the 12-year incidence of cataract surgery was 9 % (95 % CI 0–20 %), whereas it was 34 % for patients in their 7th decade (95 % CI 26–43 %, Gray's *P* = 0.02, Supplementary Fig. 2).

In summary, cataract surgery incidence varied with treatment type, tumor location, and thickness, but was not influenced by patient sex. Moreover, older age at the time of plaque brachytherapy corresponded with a higher incidence of both cataract surgery and all-cause mortality.

### 3.3 Competing risk regressions

In univariate competing risk regressions, older patient age at the time of brachytherapy (per increasing decade, continuous variable), female sex (vs. male sex, categorical variable), >5 mm thick tumors (vs. ≤5 mm, categorical variable), and iodine-125 brachytherapy (vs. ruthenium-106, categorical variable) were associated with cataract surgery. Poor BCVA of the tumor eye prior to plaque brachytherapy (LogMAR ≥1.00), anterior tumor location, and retinal detachment were not significant predictors.

In multivariate analysis, older patient age and iodine-125 retained their significance, independent of patient sex, BCVA, tumor thickness, and tumor location. Iodine-125 was the strongest predictor by far (Wald statistic 20.3).

When analyzing patients treated with ruthenium-106 and iodine-125 separately, scleral dose (which corresponds to tumor thickness) and dose rate at the tumor apex were not predictors of cataract surgery (Table 2).

**Table 2:** Uni- and multivariate competing risk regressions, subdistribution hazard ratio (exp(β_j_)) for cataract surgery after plaque brachytherapy for posterior uveal melanoma.

	β_j_	S.E.	*z*	*P*	exp(β_j_)	95 % CI	Wald
Clinicopathological, univariate							
Patient age at brachytherapy^a^	0.38	0.06	6.75	<0.001	1.46	1.31 to 1.63	45.6
Females vs. males	0.20	0.09	2.10	0.04	1.22	1.01 to 1.47	4.4
Poor BCVA^b^	0.25	0.14	1.85	0.06	1.29	0.99 to 1.68	3.4
Thick vs. thin tumors^c^	0.56	0.13	4.46	<0.001	1.75	1.37 to 2.23	19.9
Tumor location^d^	0.35	0.20	1.78	0.07	1.42	0.97 to 2.08	3.2
Retinal detachment^e^	−0.13	0.15	−0.89	0.37	0.88	0.66 to 1.17	0.8
I-125 vs. Ru-125	0.75	0.13	6.03	<0.001	2.13	1.66 to 2.72	36.4
**Clinicopathological, multivariate**							
Patient age at brachytherapy^a^	0.14	0.07	2.02	0.04	1.15	1.00 to 1.31	4.1
Females vs. males	−0.08	0.19	−0.40	0.69	0.93	0.64 to 1.35	0.2
Poor BCVA^b^	0.03	0.22	0.13	0.89	1.03	0.67 to 1.58	<0.1
Thick vs. thin tumors^c^	0.24	0.24	1.01	0.31	1.27	0.80 to 2.01	1.0
Tumor location^d^	0.33	0.23	1.44	0.15	1.39	0.89 to 2.19	2.1
I-125 vs. Ru-125	1.01	0.22	4.50	<0.001	2.75	1.77 to 4.26	20.3
**Ruthenium-106 only, univariate**							
Scleral dose^f^	<0.01	0.08	0.03	0.98	1.00	0.86 to 1.17	<0.01
Dose rate^g^	0.03	0.07	0.38	0.71	1.03	0.89 to 1.19	0.1
**Iodine-125 only, univariate**							
Scleral dose^h^	0.01	0.09	0.13	0.90	1.01	0.85 to 1.20	0.2
Dose rate^i^	−0.08	0.08	−0.9	0.34	0.93	0.79 to 1.09	0.9

a Per increasing decade.

b LogMAR ≥1.00 in tumor eye prior to plaque brachytherapy.

c Tumor apical thickness >5 mm vs. ≤5 mm.

d Tumor center anterior to the equator vs. posterior to the equator vs. posterior pole (within a 3 mm radius from the fovea, reference category).

e Exudative retinal detachment extending outside tumor margins upon slit-lamp biomicroscopy examination prior to plaque brachytherapy vs. no retinal detachment (reference category).

f Quartile 4 (≥668 Gy) vs. quartile 3 (415–667 Gy) vs. quartile 2 (312–414 Gy) vs. quartile 1 (<312 Gy, reference category).

g Quartile 4 (≥2.0 Gy/h) vs. quartile 3 (1.4–1.9 Gy/h) vs. quartile 2 (1.0–1.3 Gy/h) vs. quartile 1 (<1.0 Gy/h, reference category).

h Quartile 4 (≥317 Gy) vs quartile 3 (271–316 Gy) vs quartile 2 (211–270 Gy) vs quartile 1 (≤210 Gy, reference category).

i At the tumor apex. Quartile 4 (≥1.5 Gy/h) vs. quartile 3 (1.2–1.4 Gy/h) vs. quartile 2 (0.9–1.1 Gy/h) vs. quartile 1 (<0.9 Gy/h, reference category). BCVA, best corrected visual acuity. CI, confidence interval. Gy, gray. h, hour I-125, Iodine-125. Ru-106, Ruthenium-106. S.E., standard error.

### 3.4 Incidence versus general population sample

**Fig. 3:**
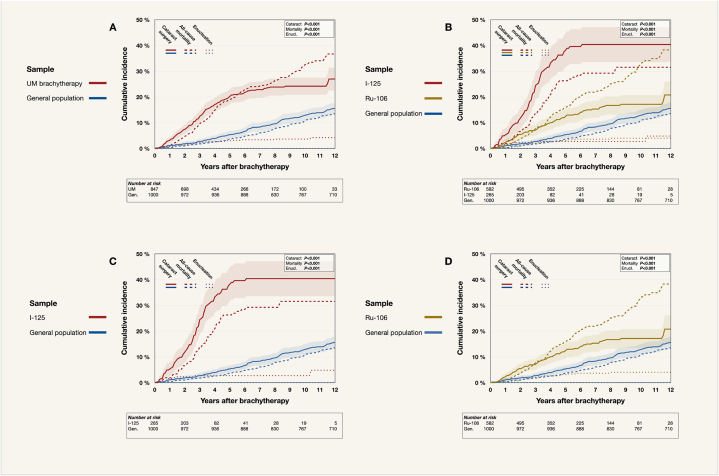
Competing risk cumulative incidence of cataract surgery, all-cause mortality, and enucleation for UM patients vs. the general population sample. A) All UM patients. B) UM patients sub distributed by radioisotope (iodine-126 or ruthenium-106). C) UM patients treated with iodine-125 only. D) UM patients treated with ruthenium-106 only. Ant., anterior. Enucl., enucleation. I-125, iodine-125. Post., posterior. Ru-106, ruthenium-106. UM, uveal melanoma. vs., versus.

The 933 UM patients had a markedly higher competing risk incidence of cataract surgery than the 1000 individuals in the general population sample, with a 12-year incidence of 27 % (95 % CI 23–31 %) versus 16 % (95 % CI 13–18 %, Gray's *P* < 0.001). As expected, UM patients also had greater incidence of all-cause mortality (*P* < 0.001, Fig. 3A).

Patients treated with Iodine-125 had significantly higher incidences of cataract surgery, all-cause mortality, and secondary enucleation than patients treated with Ruthenium-106, who in turn had greater incidences than the general population (*P* < 0.001, Fig. 3B–D).

In summary, when compared to the general population, UM patients treated with plaque brachytherapy showed a considerably higher risk of undergoing cataract surgery, especially those treated with Iodine-125. As anticipated, UM patients also displayed a higher incidence of all-cause mortality.

### 3.5 Outcomes

**Fig. 4:**
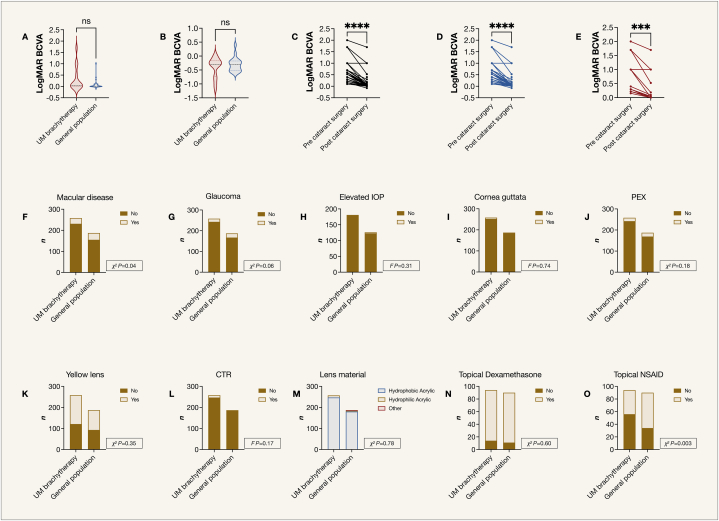
Outcomes of cataract surgery. The results were overall similar for the 172 UM patients that had undergone cataract surgery after plaque brachytherapy, and the 187 individuals from the general population sample. No endophthalmitis was observed in either group. A) The median BCVA for UM patients and individuals from the general population after cataract surgery was LogMAR 0.04 (Snellen 18/20, decimal 0.91) and 0.00 (Snellen 20/20, decimal 1.0), respectively (Mann-Whitney *U P* = 0.18). B) Median BCVA improvement, calculated as the BCVA after minus before cataract surgery, was also similar between the UM and general population sample (Wilcoxon matched-pairs signed rank *P* = 0.17). C) The median BCVA improvement for all UM patients and the general population sample combined was LogMAR 0.30 (Snellen 20/40, decimal 0.5, Wilcoxon matched-pairs signed rank *P* < 0.001). Similarly, when analyzing D) UM patients, and E) the general population separately, the BCVA improvement was identical at LogMAR 0.30 (Wilcoxon matched-pairs signed rank *P* < 0.001). F) At the time of cataract surgery, more UM patients were observed to have macular degeneration (any type, χ^2^*P* = 0.04). G) There were no statistically significant differences in rates of glaucoma (χ^2^*P* = 0.06); H) Elevated IOP (F *P* = 0.31); I) Cornea guttata (F *P* = 0.74); J) PEX (χ^2^*P* = 0.18); K) Use of yellow lenses (χ^2^*P* = 0.35); L) Use of CTR (F *P* = 0.17); M) Lens material (χ^2^*P* = 0.78); N) Or in the use of topical dexamethasone after cataract surgery (χ^2^*P* = 0.60). O) The use of topical NSAID was more common in the general population sample (χ^2^*P* = 0.003). BCVA, best corrected visual acuity. CTR, capsular tension ring. χ^2^, chi-Square test. F, Fisher's exact test. IOP, intraocular pressure. UM, uveal melanoma. NSAID, non-steroidal anti-inflammatory drugs. PEX, pseudo exfoliation syndrome.

The results of cataract surgery were overall similar for the 172 UM patients (258 minus the 86 that had undergone their cataract surgery prior to plaque brachytherapy) and 187 from the general population sample that had undergone cataract surgery. No endophthalmitis was observed in either group. The mean refractive surprise was +0.08 diopters (SD 0.59) for UM patients and −0.08 diopters (SD 0.77) in the general population (Mann-Whitney *U P* = 0.84). The median BCVA for UM patients and individuals from the general population after cataract surgery was LogMAR 0.04 (Snellen 18/20, decimal 0.91) and 0.00 (Snellen 20/20, decimal 1.0), respectively (Mann-Whitney *U P* = 0.18, Fig. 4A). Median BCVA improvement, calculated as the BCVA after minus before cataract surgery, was also similar between the UM and general population sample (Wilcoxon matched-pairs signed rank *P* = 0.17, Fig. 4B). The median difference between preoperative and postoperative BCVA for UM patients and the general population sample combined was LogMAR 0.30 (Snellen 20/40, decimal 0.5, Wilcoxon matched-pairs signed rank *P* < 0.001, Fig. 4C). Similarly, when analyzing UM patients, and the general population separately, the BCVA improvement was identical at LogMAR 0.30 (Wilcoxon matched-pairs signed rank *P* < 0.001, Fig. 4D and E). At the time of cataract surgery, more UM patients were observed to have macular degeneration (any type, χ^2^ *P* = 0.04), and those who were recorded as having macular degeneration among UM patients tended to have posteriorly located tumors: 2 of 8 (25 %) had tumors in the posterior pole; 15 of 137 (11 %) posterior to the equator; and 2 of 22 (9 %) anterior to the equator. The trend was not statistically significant (χ^2^ *P* = 0.27). There were no statistically significant differences in rates of glaucoma (χ^2^ *P* = 0.06), elevated intraocular pressure (IOP, Fisher's exact (F) *P* = 0.31), cornea guttata (F *P* = 0.74), pseudo exfoliation syndrome (PEX, χ^2^ *P* = 0.18), use of yellow lenses (χ^2^ *P* = 0.35), use of capsular tension ring (CTR, F *P* = 0.17), lens material (χ^2^ *P* = 0.78), or in the use of topical dexamethasone after cataract surgery (χ^2^ *P* = 0.60). The use of topical NSAID was more common in the general population sample (χ^2^ *P* = 0.003, Fig. 4F–O).

## 4 Discussion

In this study, we demonstrate that plaque brachytherapy is associated with an increased incidence of cataract surgery. This was particularly pronounced for those who were treated with iodine-125, with a 2.5-fold cumulative 12-year incidence versus the general population sample, whereas those who were treated with ruthenium-106 had a 1.3-fold incidence. The increased incidence associated with iodine-125 can likely be attributed to the slower dose fall-off of gamma radiation. This leads to higher doses being delivered to the lens for equal doses to the tumor apex. Such intrinsic property underscores the importance of weighing therapeutic benefits against potential ocular complications critically.

Given these findings, future treatment plans could integrate methods to mitigate this effect. For example, in appropriate cases, opting for brachytherapy with a beta-emitting isotope like ruthenium-106 over a gamma-emitting source might be beneficial. We have previously shown a non-inferior survival rate for patients with tumors ≥5.5 mm thick when treated with ruthenium-106 [14]. It's imperative to note that while thicker tumors are more likely to be treated with iodine-125—a stronger predictor for cataract surgery—the different intrinsic properties of beta and gamma radiation played a substantial role. Tumor thickness >5 mm was not identified as an independent risk factor in our study due to these radiation property distinctions.

On the other hand, cataract surgery is a quick procedure associated with limited risks for local and systemic complications, and one cannot justify an increased risk for tumor recurrence with a decreased risk for cataract [27,28]. In another previous study, tumor thickness >4 mm has been linked to an increased risk for treatment failure after plaque brachytherapy with ruthenium-106, which in turn could have negative long-term consequences for the metastatic risk [[29], [30], [31], [32]].

When assessing the overall condition of the eyes during the time of cataract surgery and the subsequent outcomes, we found them to be similar between the plaque brachytherapy recipients and the general population. There were no significant disparities in aspects like refractive surprise, median post-surgery BCVA, median BCVA enhancement, or in occurrences of conditions like glaucoma, elevated IOP, cornea guttata, PEX, or the application of treatments like yellow lenses, capsular tension rings, or dexamethasone.

The differences in rates of macular degeneration and use of topical NSAID is noteworthy and could be explored further. Radiation maculopathy is one of the most common causes of visual loss following radiotherapy, and typically develops when radiation exposure extends beyond tissue tolerance [33,34]. There is a possibility that certain patients with radiation maculopathy might have been diagnosed with macular degeneration by the cataract surgeon. This inference is perhaps suggested by the non-significant trend fore more posteriorly located tumors in this subset. Yet, regardless of its specific etiology, this condition did not result in inferior BCVA outcomes for the plaque brachytherapy group. Interestingly, the increased use of topical NSAID in the general population presents a conundrum, and the reason for this difference is elusive. One might only speculate in a random coincidence, or perhaps a hesitancy among cataract surgeons to prescribe specific medications to oncology patients.

Patient sex was not associated with the incidence of cataract surgery, which complements our previous observation of similar rates of tumor relapses, repeated brachytherapy, enucleation-free survival, and melanoma-related mortality [35]. Others have found an increased risk in males for complications after plaque brachytherapy, including the development of cataract [20].

Two previous studies with shorter-follow up and smaller patient samples have reported a 5-year incidence of cataract surgery of 12 % (95 % CI 9–15 %) using the Kaplan-Meier product-limit method, and an 8-year competing risk incidence of just over 50 %, respectively [18,20]. Both of these previous studies examined patients treated exclusively with iodine-125 brachytherapy, and one reason for the large difference in observed incidence is marked dissimilarities in mean tumor size. In both studies, increased tumor thickness was identified as a risk factor for cataract. This observation was herein nuanced by the different intrinsic properties of beta and gamma radiation.

Another reason for differences between studies may be variations in statistical methods. The Kaplan-Meier method was originally developed to assess all-cause mortality and assumes that censored individuals are still at risk of the event of interest, i.e., death. In analysis of other types of events, such as cataract surgery or death from a specific cause, the Kaplan-Meier is less suitable as there will be competing risk, e.g., enucleation or death from any other cause, that render patients immune to the event of interest. Therefore, competing risk analyses is preferable in these situations.

Other strengths of this study include the comparisons with a large general population sample, rendering a post-hoc power of >99.5 %, which allows for a comprehensive analysis of the differences in incidence and outcomes. Clearly, cataract is no rarity in a random sample of individuals of the same sex and age from the general population. This is further underlined by the fact that patient age at the time of plaque brachytherapy was a predictor of cataract surgery, with a 15 % higher risk for every increased decade of age, independent of patient sex, BCVA, tumor thickness, tumor location, and radioisotope. This could be interpreted as the contribution from natural aging on the risk for cataract development, which will affect UM patients and the general population to an equal extent.

### 4.1 Importance for patients

Cataract can have a significant impact on daily life. In our study, we observed that the mean BCVA prior to undergoing cataract surgery was at a level of LogMAR 0.53. Translated, this approximates to a Snellen reading of 20/60 or a decimal measure of 0.32. At this level, individuals may find themselves significantly impaired in routine activities. Tasks such as driving, reading, watching television, and even navigating public transportation systems can become increasingly challenging. The quality of life may degrade, with some activities becoming nearly impossible, depending on the condition of the other eye and overall visual fields.

While cataract surgery is generally a straightforward procedure with a minimal risk profile, it's vital to remember that it remains an invasive intraocular operation. This very nature of the procedure may make certain patients hesitant. Some might prefer to postpone the surgery if feasible or, in rare cases, decide against undergoing it altogether. Therefore, it's essential that patients receive detailed information about cataract risks and other potential complications before undergoing plaque brachytherapy. Such risks should be considered when evaluating different radioisotope options.

## 5 Limitations

There are several limitations to this study. Firstly, interventions were not randomized. This implies that the difference in outcomes after plaque brachytherapy with ruthenium-106 and iodine-125 could have been influenced by factors not fully accounted for. Secondly, even though we randomly drew a comparison sample of 1000 individuals from the general population, and there were no significant differences in age or sex between these individuals and the UM patients, we cannot rule out that there were other differences in baseline characteristics that modified the risk for cataract surgery. E.g., a UM patient will typically undergo regular follow-up visits with eye examinations, potentially increasing the likelihood that a cataract will be detected and that a referral for surgery will be sent compared to a person who does not undergo regular eye exams. Lastly, although our data encompasses all patients treated with plaque brachytherapy in Sweden, considering that we are the only institution in the country that offers this treatment, and the Swedish Cataract Register is estimated to have covered 93 % of cases from 2010 to 2021, we cannot discount the possibility that some cataract surgeries in the UM or general population sample were not accounted for [22].

## 6 Conclusions

Overall, plaque brachytherapy is clearly associated with an increased incidence of cataract surgery, with a 2.5-fold incidence for patients treated with iodine-125 versus the general population sample, and 1.3-fold incidence for those treated with ruthenium-106. The second strongest risk factor for cataract surgery was patient age at the time of plaque brachytherapy. Post cataract-surgery outcomes were similar between UM patients and the general population. Differences in macular degeneration rates and the use of topical NSAIDs between the groups warrant further investigation.

## Ethics statement

The study adhered to the tenets of the Declaration of Helsinki, and approval was obtained from the Swedish Ethical Review Authority (reference 2022-00930-02). Informed consent was waived because this was a retrospective chart review that did not influence patient management (i.e., treatment, testing, follow-up, or information to patients), did not include any analyses of biological tissues, and was based on already collected data.

## Data availability statement

The data supporting the findings of this study have not been deposited in a publicly accessible repository, owing to restrictions related to the sharing of sensitive data.

## CRediT authorship contribution statement

**Viktor Gill:** Writing – review & editing, Validation. **Gustav Stålhammar:** Writing – review & editing, Writing – original draft, Visualization, Validation, Supervision, Software, Resources, Methodology, Investigation, Funding acquisition, Formal analysis, Data curation, Conceptualization.

## Declaration of competing interest

The authors declare the following financial interests/personal relationships which may be considered as potential competing interests:Gustav Stalhammar reports financial support was provided by the 10.13039/501100007687Swedish Society of Medicine. Gustav Stalhammar reports financial support was provided by the Swedish Eye Foundation. Gustav Stalhammar reports financial support was provided by Region Stockholm. Gustav Stalhammar reports financial support was provided by the 10.13039/501100002794Swedish Cancer Society. Gustav Stalhammar reports financial support was provided by Crown Princess Margareta's Working Group for the Visually Impaired. Gustav Stalhammar reports financial support was provided by Carmen and Bertil Regnér Foundation for Eye Disease Research. If there are other authors, they declare that they have no known competing financial interests or personal relationships that could have appeared to influence the work reported in this paper.
