# Supplementary material for: Incidence, risk factors and outcomes of cataract surgery after plaque brachytherapy for posterior uveal melanoma
Source: Heliyon. 2023 Dec 9;10(1):e23447. doi: 10.1016/j.heliyon.2023.e23447 (PMC10761564; doi:10.1016/j.heliyon.2023.e23447)
Supplement: Multimedia component 1 [file mmc1.pdf]

Supplementary Material to

Incidence, Risk Factors and Outcomes of Cataract Surgery after Plaque Brachytherapy for Posterior Uveal Melanoma

Viktor Gill, M.D.[1,2], Gustav Stålhammar, M.D. Ph.D.[2,3]

[1]Department of Pathology, Västmanland Hospital Västerås, Västerås, Sweden.
[2]Department of Clinical Neuroscience, Division of Eye and Vision, Karolinska Institutet, Stockholm, Sweden
[3]St. Erik Eye Hospital, Stockholm, Sweden

Supplementary table 1. Number of cataract surgeries registered in the Swedish National Cataract Register 2010–2022

Year	Cataract surgeries, *n*
2010	88 695
2011	92 752
2012	94 913
2013	108 501
2014	111 228
2015	113 449
2016	119 818
2017	124 811
2018	129 309
2019	131 245
2020	115 828
2021	133 707
2022	151 727
**Sum**	**1 515 983**

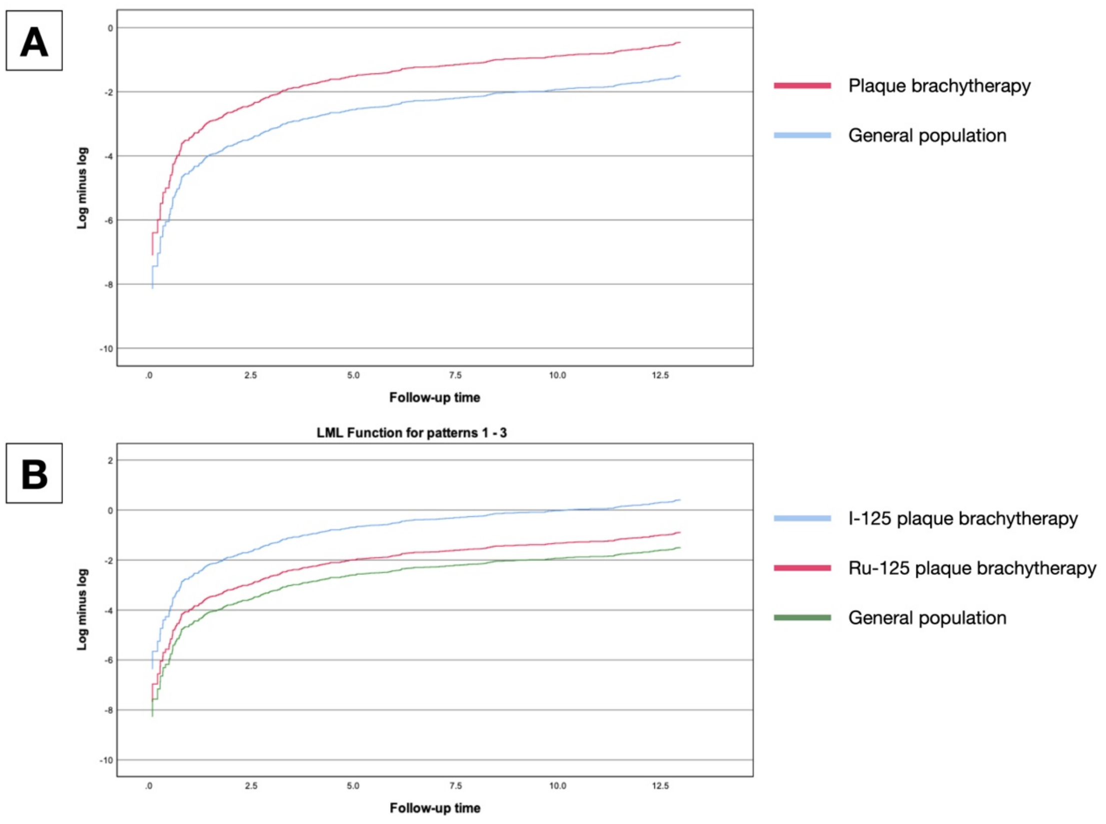

Supplementary Figure 1. Log-minus-log survival curves illustrating the proportional hazards assumption. A) Comparison between uveal melanoma patients treated with plaque brachytherapy and the general population. B) Uveal melanoma patients treated with plaque brachytherapy, subdivided into those treated with ruthenium-106 (Ru-106) and iodine-125 (I-125), compared with the general population.

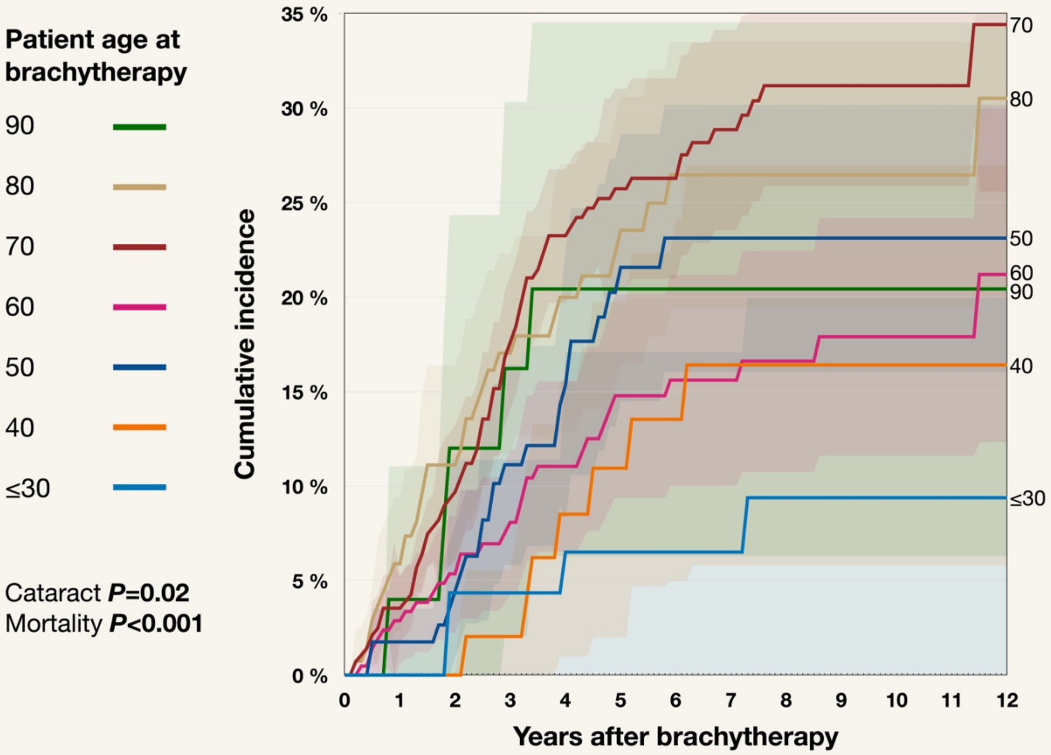

Number at risk							
≤30	25	21	18	14	9	5	2
40	53	50	36	26	18	15	4
50	114	102	70	41	30	16	7
60	210	171	108	70	46	21	9
70	283	229	137	83	51	32	6
80	136	105	52	24	14	10	5
90	25	19	13	8	4	1	1

Supplementary figure 2. Cumulative incidence of cataract surgery by patient age at the time of plaque brachytherapy. For patients ≤30 years at the time of brachytherapy, the 12-year incidence of cataract surgery was 9% (95% CI 0–20%), whereas it was 34% for patients in their 7th decade (95% CI 26–43%, Gray's *P*=0.02).
